# Analytical Methods for Assessing Thiol Antioxidants in Biological Fluids: A Review

**DOI:** 10.3390/molecules29184433

**Published:** 2024-09-18

**Authors:** Iuliia A. Poimenova, Madina M. Sozarukova, Daria-Maria V. Ratova, Vita N. Nikitina, Vladislav R. Khabibullin, Ivan V. Mikheev, Elena V. Proskurnina, Mikhail A. Proskurnin

**Affiliations:** 1 Analytical Chemistry Division, Department of Chemistry, Lomonosov Moscow State University, 1-3 Leninskie Gory, 119234 Moscow, Russia; poymenovajul@gmail.com (I.A.P.); s_madinam@bk.ru (M.M.S.); darmarrat@gmail.com (D.-M.V.R.); niki-vita@yandex.ru (V.N.N.); vladhab1995@gmail.com (V.R.K.); 2 Kurnakov Institute of General and Inorganic Chemistry, Russian Academy of Sciences, 117901 Moscow, Russia; proskurnina@gmail.com; 3 Federal State Budgetary Institution of Science Institute of African Studies, Russian Academy of Sciences, Spiridonovka St., 30/1, 123001 Moscow, Russia; 4 Laboratory of Molecular Biology, Research Centre for Medical Genetics, 1 Moskvorechye St., 115522 Moscow, Russia

**Keywords:** analysis of thiols, biological fluids, redox status, analytical methods, biothiols

## Abstract

Redox metabolism is an integral part of the glutathione system, encompassing reduced and oxidized glutathione, hydrogen peroxide, and associated enzymes. This core process orchestrates a network of thiol antioxidants like thioredoxins and peroxiredoxins, alongside critical thiol-containing proteins such as mercaptoalbumin. Modifications to thiol-containing proteins, including oxidation and glutathionylation, regulate cellular signaling influencing gene activities in inflammation and carcinogenesis. Analyzing thiol antioxidants, especially glutathione, in biological fluids offers insights into pathological conditions. This review discusses the analytical methods for biothiol determination, mainly in blood plasma. The study includes all key methodological aspects of spectroscopy, chromatography, electrochemistry, and mass spectrometry, highlighting their principles, benefits, limitations, and recent advancements that were not included in previously published reviews. Sample preparation and factors affecting thiol antioxidant measurements are discussed. The review reveals that the choice of analytical procedures should be based on the specific requirements of the research. Spectrophotometric methods are simple and cost-effective but may need more specificity. Chromatographic techniques have excellent separation capabilities but require longer analysis times. Electrochemical methods enable real-time monitoring but have disadvantages such as interference. Mass spectrometry-based approaches have high sensitivity and selectivity but require sophisticated instrumentation. Combining multiple techniques can provide comprehensive information on thiol antioxidant levels in biological fluids, enabling clearer insights into their roles in health and disease. This review covers the time span from 2010 to mid-2024, and the data were obtained from the SciFinder^®^ (ACS), Google Scholar (Google), PubMed^®^, and ScienceDirect (Scopus) databases through a combination search approach using keywords.

## 1. Introduction

In recent decades, the field of medical science has shifted its focus toward the examination of redox status due to its pivotal role in health and disease. Redox status, denoting the equilibrium between reduced and oxidized molecules within the organism, assumes a central role in regulating cellular functions and signaling pathways. It is widely acknowledged that the thiol/glutathione disulfide (GSH) ratio represents a critical determinant of redox status. GSH is a tripeptide comprising cysteine (Cys), glycine (Gly), and glutamic acid (Glu) and assumes a prominent role as a primary antioxidant, thoroughly mitigating oxidative stress and maintaining the cellular redox equilibrium [1,2,3].

The strong influence of redox status extends to thiol-dependent redox signaling pathways, intricately regulated by a group of proteins and enzymes [4,5]. The complex dynamics of redox status significantly impact the activity of these pathways, which govern a multitude of physiological processes. The supreme importance of these pathways becomes conspicuous when considering pathological conditions characterized by disrupted or altered redox signaling. Such diseases include carcinogenesis, inflammation, and ischemia, which together cover a wide range of clinical disorders [6,7,8,9,10]. For instance, the dysregulation of the Nuclear Factor Kappa B (NF-κB) signaling pathway is evident in chronic inflammatory conditions like rheumatoid arthritis, inflammatory bowel disease, and asthma [11,12]. Furthermore, aberrant NF-κB signaling is strongly linked to the development and progression of cancer, while autoimmune disorders such as lupus and multiple sclerosis also exhibit disturbances in this pathway [13,14]. The levels of cysteine were reduced in cases of acute ischemic stroke, and no variations in cysteine levels were observed among different stroke subtypes [15].

Diseases associated with oxidative stress, such as Alzheimer’s and Parkinson’s, often exhibit impaired Nrf2 (nuclear factor erythroid 2-related factor [16]) signaling, which facilitates cancer cell survival and resistance to chemotherapy [17,18,19]. Changes in protein tyrosine phosphatase (PTP) activity are associated with the development of insulin resistance and diabetes, and disrupted PTPs play a role in the onset of cancer [19]. Irregularities in mitogen-activated protein kinase (MAPK) signaling are a common feature in multiple cancer types and are also implicated in Alzheimer’s disease, as well as contributing to the development of certain heart diseases [20,21]. It is crucial to recognize that the influence of redox-dependent signaling extends far beyond these specific diseases, including a broad spectrum of medical conditions.

Within the landscape of molecular regulation, proteins that contain thiol groups, such as human serum albumin (HSA), play important roles. These proteins are not passive participants; rather, they orchestrate the functioning of redox signaling pathways. Their activity and, by extension, regulation of vital cellular processes hinge on the finely tuned equilibrium maintained by the thiol/disulfide ratio of glutathione [22,23]. HSA interacts with various proteins and receptors on cell membranes, which can trigger diverse signaling cascades within cells, influencing processes such as proliferation, apoptosis (programmed cell death), and protein synthesis [24,25]. Hence, to gain profound insights into the progression mechanisms of diverse pathologies and to devise precise therapeutic strategies, it is imperative to conduct a comprehensive and quantitative evaluation of the thiol pool within clinical specimens.

The simultaneous, accurate, and reliable determination of both the oxidized and reduced forms of thiols in biological samples, including the assessment of redox status, continues to be a pressing issue in analytical chemistry. It should be mentioned that pioneering reviews were devoted to the complete assessment of the reduced form of glutathione (GSH, γ-L-glutamyl-L-cysteinylglycine). The primary methods used since the 2000s have been HPLC and CE for the separation and determination of GSH and metabolites. However, attempts have been made to reduce and improve the detection limit of UV–Vis and fluorescence spectroscopies [26]. The reviews published over the last decade on this subject have focused on the following:

- 2010–2015: Monitoring protein thiol redox states [27] and determining low-molecular-weight biothiols in human plasma and blood [28];
- 2015–2020: Making breakthroughs using carbon dot- and graphene quantum dot-based fluorescent sensors for biothiol analysis [29] and assessing glutathione and glutathione disulfide in pharmaceuticals and biological fluids [30];
- 2020–now: Evolving thiol sensing, from current methods to nanoscale contributions [31], by increasing the versatility and effectiveness of chromatography and mass spectrometry in detecting sulfhydryl compounds in biological samples [32].

All of these reviews show multiple attempts to solve these issues separately using methods such as spectroscopy, chromatography, mass spectrometry, electroanalytical methods, and various other methods. To accelerate the comparison and gain a comprehensive understanding of potential and noteworthy applications in this field, our review involves a range of approaches. However, some works may overlook or insufficiently discuss the biochemistry of thiol groups and the crucial sample preparation step, which is vital due to the sulfhydryl group’s (–SH) high reactivity. Protecting the sulfhydryl group from oxidation during sample handling, storage, and analysis is essential but can increase the analysis duration, labor intensity, and demand for sensitive and assay-specific, specialized equipment and expertise [30,33]. This review is primarily dedicated to the various analytical methodologies used for the analysis of thiol and disulfide derivatives. To gain a better understanding of how biothiols are regulated, it is essential to familiarize oneself with the recent and pioneering research in the field dealing with antioxidant activity and capacity [34] and the functions of antioxidants [35].

Given the constant updates of information on thiols’ biochemical, kinetic, and chemical properties, it is crucial to consider various up-to-date options for thiol determination using analytical chemistry methods, along with sample preparation methods and associated challenges. The relevance of these procedures in addressing clinical and laboratory diagnostic issues should be equally emphasized. Exploring the potential of chemiluminometry as an alternative to conventional analytical chemistry methods holds promise and merits a separate investigation and comparative analysis. As the field progresses, a comprehensive approach to thiol determination becomes essential for achieving accurate and meaningful results to understand the roles of thiols in biological systems.

This review primarily concentrates on prevalent methodologies used for the quantification of thiol antioxidants, particularly glutathione, cysteine, and albumin, in blood plasma, a prominent sample type in clinical and laboratory diagnostics. It highlights current trends and potential innovations in analytical chemistry related to this issue. Notably, it emphasizes chemiluminescent techniques as a highly promising path within this domain.

This study mostly includes papers from 2010 to mid-2024 that were obtained by searching the SciFinder^®^ (ACS), Google Scholar (Google), PubMed^®^, and ScienceDirect (Scopus) databases, querying a combination of keywords. Readers will find the entire list of abbreviations in Abbreviations part, which is provided for their convenience.

## 2. Redox Balance: From Antioxidants to Oxidative Stress and Signaling

After Howard M. Shapiro introduced the concept of the redox balance [36], Barry Halliwell and John M.C. Gutteridge discussed the definition and measurement of antioxidants in biological systems [37], and Helmut Sies defined the “oxidative stress” concept [38], followed by many works concerning redox regulations and oxidative stress quantification. The concept of redox status has evolved from foundational insights into chemical reactions to a pivotal concept in biochemistry and medical research, holding profound implications for understanding health and disease. Redox status represents the balance between reduction and oxidation in biological entities. The essence of redox reactions lies in the application of thermodynamic concepts [39]. To begin with, it is essential to understand the concept of energy transfer, such as in the respiratory chain, where the direction of electron movement is determined by changes in free (Gibbs) energy [40]). This principle holds great significance.

Specifically, in human biochemistry, it encapsulates the collective electron flux and redox reactions transpiring in cells, tissues, or organisms. While the general term includes an array of redox-active molecules, it is predominantly employed to denote the equilibrium between the reduced and oxidized states of glutathione (GSH/GSSG), occasionally extending to include redox couples like NAD^+^/NADH and NADP^+^/NADPH in biological systems [41]. Glutathione, a tripeptide constituted by glutamate, cysteine, and glycine, is endowed with a thiol group (–SH), allowing it to function as both a reductant and a nucleophile. The delineation of the glutathione transformation cycle holds significant merit. Glutathione is one of the most abundant antioxidants within cells, and its cycle occupies a central position within the cellular antioxidant defense system, governing redox reactions within biological domains owing to its comparatively low standard redox potential [42]. For the reaction GSSG + 2H^+^ + 2e^−^ → 2GSH, the standard (midpoint) potential [43] is defined as $E^{0^{\prime }}=E_{h_{GSH/GSSG}}$ = −240 mV 2GSH/GSSG at 25 °C, pH 7.0 [44]. Antioxidants and oxidants interact complexly, mitochondria regulate redox through electron passage in the respiratory chain, and older antioxidant enzymes react. Analyzing thiol antioxidants alone is insufficient for understanding pathological conditions. However, this analysis is a crucial issue in analytical chemistry methodology.

### 2.1. Signaling

Signaling is a sequence of events that affect genes and cause their activation or repression. Therefore, it is necessary to outline the main points of redox signaling and which processes depend on it, such as carcinogenesis and inflammation. The central hub of redox signaling is appropriately identified as a cycle of transformations involving sulfur-containing proteins, instigated by hydrogen peroxide and facilitated by reducing agents such as thioredoxins (Trxs) and glutaredoxins (Grxs, dynamic thiol–disulfide oxidoreductases) [45], along with glutathione [46].

It is evident that changes in redox status, specifically concerning reduced and oxidized glutathione, are correlated with disturbances in H_2_O_2_-dependent redox signaling due to cysteine oxidation. At modest concentrations, hydrogen peroxide can activate transcription factors, like NF-κB and AP-1 (activator protein-1), and enzymes within signaling pathways, including tyrosine kinases, tyrosine phosphatases, and MAPKs, thus inducing eustress [46]. NF-κB translocates to the nucleus, stimulating the expression of genes associated with inflammation, immune responses, and cell survival [47,48]. Analogously, heightened hydrogen peroxide levels can activate AP-1 by promoting the phosphorylation and activation of its constituents, such as c-Jun and c-Fos (proto-oncogene-encoded proteins [49]), through MAPKs [48,50]. Elevated H_2_O_2_ levels may oxidize critical cysteine residues in tyrosine kinases, resulting in their inactivation or modified activity. This impaired functionality disrupts downstream signaling cascades that play pivotal roles in essential cellular processes such as growth, proliferation, and differentiation [51].

Within the domain of redox signaling and pathological conditions, carcinogenesis and inflammation stand as paramount processes that are profoundly impacted. As previously outlined (refer to the Section 1), deviations or modifications in redox signaling are common in clinical scenarios. Consequently, a disruption in redox signaling makes up an inevitable phase in the progression of a vast array of pathologies, clearly reflected in the redox status, particularly concerning the glutathione–glutathione disulfide equilibrium. Recent publications emphasize that evaluating alterations in specific thiol pools associated with individual diseases can offer more pertinent and dependable insights into pathological progression in a person [52]. Extensive research, particularly focusing on cardiovascular ailments and their related thiol/disulfide ratios, has produced substantial data. For instance, an elevated GSH/GSSG ratio in the plasma of non-smokers has emerged as an independent predictive factor for early atherosclerosis [53]. Moreover, heightened cysteine disulfide levels are linked to endothelial dysfunction [54]. The cysteine disulfide/GSH ratio escalation in plasma among individuals with coronary heart disease signals a higher risk of mortality within a five-year timeframe [55]. Depending on the type and stage of cancer, distinct modifications in thiol pools can serve as indicative markers of the disease. An elevated Cys concentration is associated with a heightened risk of colorectal cancer [56], while an inverse correlation is observed in breast, esophageal, and gastric cancers [57,58]. Although the exact role of thiols in the development of neurodegenerative diseases is yet to be fully elucidated, substantial evidence links increased plasma homocysteine (Hcys) levels to a heightened likelihood of dementia and Alzheimer’s disease among patients [59]. As highlighted in recent publications, the assessment of oxidative stress is intricately tied to the characteristics of the biomarker employed for its estimation and the level of standardization achieved in the measurement methodologies [60,61].

### 2.2. Primary Reactions of Thiols in Biological Systems

Helmut Sies defined oxidative stress as “an imbalance favoring oxidants over antioxidants, resulting in disruptions in redox signaling, control, and/or molecular damage” [42]. The oxidizing agents referred to in this definition comprise reactive oxygen species (ROS) generated during the metabolism of aerobic organisms, including superoxide anion radical (^•^O_2_^−^), singlet oxygen (^1^O_2_), hydrogen peroxide (H_2_O_2_), and hydroxyl radicals (^•^OH), among others. The classification of oxidative stress into oxidative eustress (controlled ROS exposure targeting specific entities) and oxidative distress (excessive ROS exposure affecting non-specific entities) are discussed in [62,63]. Oxidative eustress occurs in regulated cellular redox signaling, while oxidative distress disrupts these signaling pathways. Redox signaling, a pivotal aspect of cellular communication, involves a sequence of events influenced by redox (reduction–oxidation) reactions. These reactions exert influences on genes, either activating or inhibiting them, and significantly contribute to diverse physiological and pathological processes. ROS assume a critical role in thiol-based modifications of target proteins, exerting influences on a range of signaling pathways. The thiol groups (–SH) in proteins, particularly cysteine residues, prove highly susceptible to oxidation by ROS, resulting in reversible or irreversible modifications that can reshape protein structure and function. This perspective is illustrated in the hydrogen peroxide-dependent and superoxide anion radical-dependent pathways, where signaling molecules (H_2_O_2_ and ^•^O_2_^−^, respectively) interact with sulfur-containing proteins (see below) [64]. Additionally, with the generation of ^•^O_2_^−^, the concurrent effective formation of nitric oxide (NO) and peroxynitrite (ONOO^−^) takes place, leading to the nitration of tyrosine residues in proteins, instigating further functional modifications [65].

Typically, thiolate ions (RS^−^) participate in reactions, and due to their negative charge, they exhibit stronger nucleophilic properties compared to the protonated form. In particular, in an alkaline environment characterized by a consistent pH level (the normal pH range is approximately 7.35 to 7.45, aligning with the average acidity of blood plasma at pH 7.4) [66], thiols with lower p*K*_a_ values will be the most reactive. The nucleophilic activity of the cysteine residue toward specific substrates within proteins is determined by the structural surroundings of the sulfhydryl group [67]. The reactivity of the non-ionized sulfhydryl group intensifies during the shift from model solutions to biological samples. During the lysis of biological entities in an acidic environment, the (–SH) group is indeed oxidized owing to the liberation of ROS from biological matrices [33].

**Figure 1:**
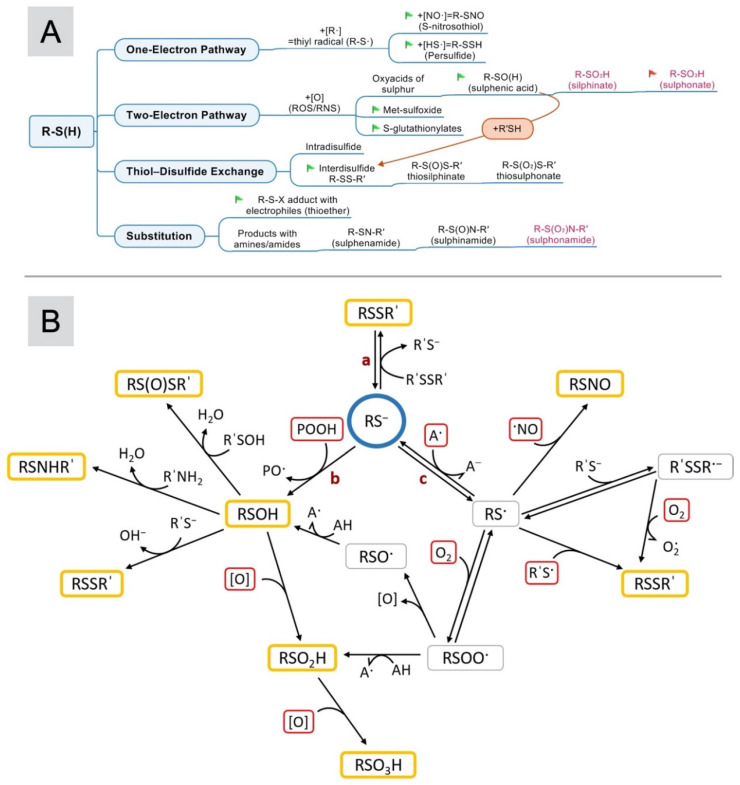
(**A**) The complete scheme of reactions that generate oxidation products of thiols in biological systems. The green flags indicate reversible reactions, and the red flag indicates an irreversible reaction. Text highlighted in fuchsia refers to products that may not occur in biological objects. (**B**) The oxidation of the thiol group of albumin [25]. The blue and red frames contain the reagents, and the yellow structures include the products. Adapted with permission from [25]. Copyright 2024, Elsevier.

Thiols play a fundamental role in numerous redox reactions within the biological system. Both low- and high-molecular-weight thiols undergo free-radical oxidation through a similar mechanism, as depicted in Figure 1. Interaction with ROS and reactive nitrogen species (RNS) results in the formation of sulfenic acids (R–SOH). Upon further oxidation, these sulfenic acids are swiftly converted into sulfinic (R–SO_2_H) and sulfonic (R–SO_3_H) acids. Sulfonic acid represents the end product of free-radical oxidation, while the formation of sulfenic and sulfinic acids is a reversible process. Thiol interaction with disulfides within the body leads to disulfide exchange reactions, yielding both intra- (R–SS) and intermolecular (R–S–S–R) disulfides [68]. Disulfide formation can also occur through reactions involving sulfenic acid, an intermediate product of ROS oxidation. Some of these reactions are reversible, while others are irreversible [69]. *S*-nitrosylation of thiols is facilitated by nitric oxide [52]. The intricacies of free-radical oxidation mechanisms can be explained through the study of albumin. Recent advancements in albumin preparation and separation techniques, along with up-to-date methodologies for its determination using spectroscopic and electrochemical methods, were described in detail in [70].

Figure 1B illustrates the chemistry of the sulfhydryl group present in this protein. In Figure 1B direction ***a***, the thiolate ion interacts with disulfides (R′SSR′) through reversible thiol–disulfide exchange reactions. The nucleophilic attack of a thiolate anion leads to an electron-pair exchange, i.e., the formation of a covalent disulfide bond. Disulfide formation and disintegration mainly depend on kinetic factors, as well as on the availability of electron donors and acceptors, which determine the total redox potential of the biological environment (E_h_) [71].

Direction ***b*** shows the oxidation of thiol by the two-electron mechanism (two-electron oxidation) under the action of hydroperoxide (POOH) to form a sulfenic acid (RSOH). Sulfenic acids are usually unstable and are recycled by reactions with other thiols to form disulfides. Typical two-electron oxidizing agents are organic and inorganic hydroperoxides and peroxy-, hypoalgesic, and halogen-amines [72]. Superoxide anion radicals can lead to the oxidation of thiols through both two-electron and one-electron mechanisms. Computer modeling of superoxide anion radical reactions has shown that two-electron oxidation predominates [73]. The one-electron mechanism is dominant in exceptional cases: for example, in hydrophobic environments [74].

Thiols can also be oxidized by a single electron (a one-electron mechanism) with the formation of highly reactive thiol radicals (RS^•^) (direction ***c***). Thiol radicals react with each other to form disulfides or with thiols to form disulfide anion radicals (R′SSR^•−^). Such R’SSR^•−^ radicals can reduce oxygen to ^•^O_2_^−^. In addition, thiol radicals can react with oxygen to form peroxyl (RSOO^•^) and other secondary radicals. The peroxyl radicals are oxidized to sulfinic (RSO_2_H) and sulfonic (RSO_3_H) acids. These compounds are considered end products and are not subject to reduction. However, there is evidence that the enzymatic reduction of sulfinic acid is possible [75]. The mechanism of one-electron oxidation is realized by the action of such particles as OH^•^, CO_3_^•−^, NO_2_^•^, and RSOO^•^ on a thiol [72].

Turning to the antioxidant properties of blood plasma, the mechanisms underlying the implementation of its antioxidant protection are complex and have not yet been thoroughly studied. This phenomenon arises from the collaborative action of antioxidants and can be exemplified through the role of glutathione. Glutathione, a vital thiol, actively takes part in the regeneration of ascorbic acid. In turn, ascorbic acid is pivotal in the regeneration of α-tocopherol from its radical forms [76]. A hierarchy determines the order in which antioxidants enter oxidation reactions [52]. As a result of this intricate organization of the antioxidant system at multiple levels, relying on a single redox couple of thiols for assessing the plasma redox state proves to be inadequately informative. Another challenge arises in the development of effective antioxidant therapies. For instance, administering antioxidants in the treatment of individuals with schizophrenia has not yielded the anticipated efficacy [77]. Using the thiol antioxidant *N*-acetylcysteine, typical in pharmacology, to treat mice with lung cancer and melanoma led to tumor overgrowth [78].

The activity of each antioxidant depends on its prevalence in the plasma and reactive availability, i.e., its structure. It is a recognized paradox that uric acid, principally a low-molecular-weight antioxidant, assumes prooxidant attributes upon surpassing a specific threshold [79,80]. In addition, in biological systems, thiol–disulfide exchange reactions are not in equilibrium [25]. This shows that the thiol redox status is determined to a greater extent by individual kinetics rather than the thermodynamic parameters for each compound. Six primary kinetic factors that impact the thiol redox status of blood plasma have been identified. These include the rates of the following processes:

- Thiol–disulfide exchange reactions [81];
- Oxidation by ROS and probable reduction processes [82];
- The extracellular enzymatic degradation of glutathione [83];
- Transport between the plasma department and cells, primarily through erythrocytes and endothelial cells [84];
- Liver release of thiol-containing molecules [85];
- Intracellular metabolism [25,52].

As a result, the concentrations of reduced thiols may undergo uneven and/or divergent modifications during the advancement of a specific ailment and the aging process [52,86,87]. Markers of structural oxidative damage and many procedures for the determination of antioxidant biomarkers in biological fluids have been reviewed in a book chapter [88].

### 2.3. Laboratory Assessment of Redox Status

Blood encompasses a comprehensive array of key participants in redox metabolism, including low-molecular-weight thiols (glutathione, L-cysteine, homocysteine, and some others) and mercaptoalbumin. These components form the thiol pool within blood plasma and are also involved in redox homeostasis, as summarized in Table 1.

The interaction of ROS with thiols initiates the formation of intermolecular and intramolecular disulfide bonds, as illustrated in Figure 1. Thus, when addressing thiol redox homeostasis, the term “reduced form” signifies the protonated state of the sulfhydryl group, while the “oxidized form” generally represents the formation of disulfide compounds.

**Figure 2:**
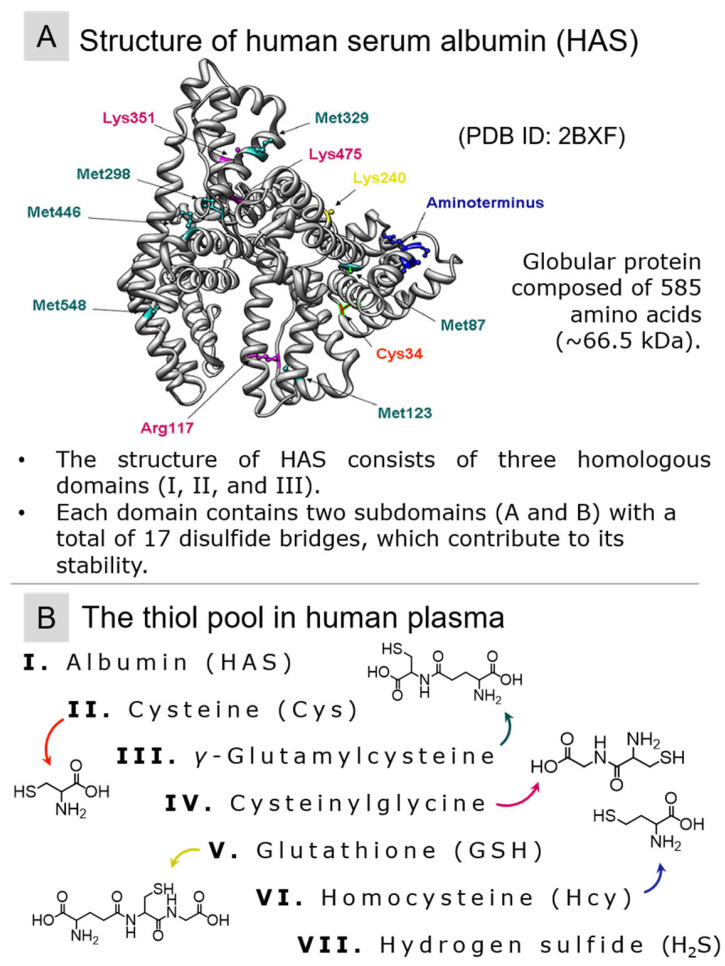
(**A**) The structure of human serum albumin (PDB ID: 2BXF) and (**B**) the main thiols with their chemical formulae belonging to the pool of blood plasma.

A notable blood participant in redox homeostasis is human serum albumin (HSA, M = 66,348 Da) (refer to Figure 2A), with an average plasma content ranging from 35 to 52 g/L (ca. 525–780 µM) [87]. At neutral pH, this functional group is protonated (with an approximate p*K*_a_ value of 8) [25]. When the sulfhydryl group of albumin is protonated, it is referred to as mercaptoalbumin. Thiol species in blood plasma are depicted in Figure 2A.

From the preceding discussion, evaluating the redox status of blood plasma stands as a highly pertinent procedure for both clinical laboratory practice and biophysical investigations. Notably, thorough examination often focuses on redox couples associated with specific compounds, notably Cys or glutathione (GSH), given their substantial impact on the reduction potential (E_h_) of blood plasma [89].

## 3. Methods and Procedures

### 3.1. Methods for Monitoring Thiol Redox Status

**Figure 3:**
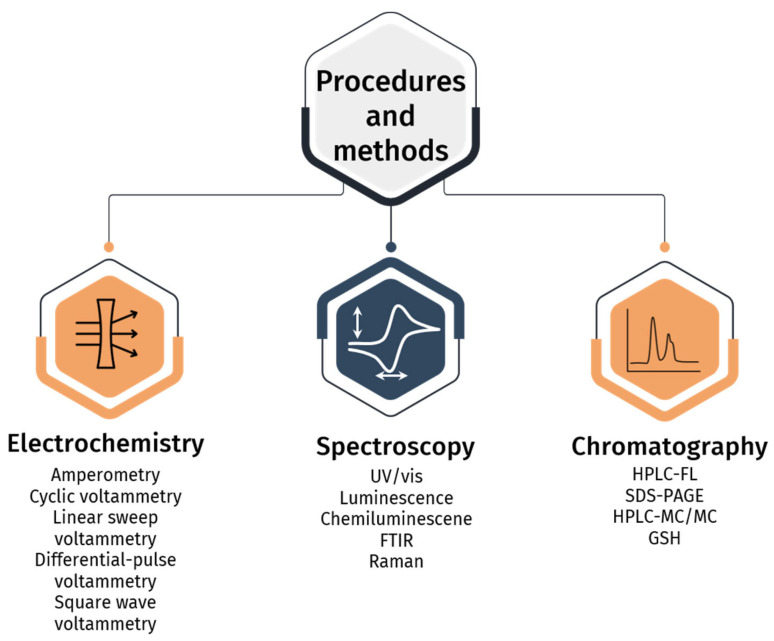
Analytical techniques mentioned in Section 3.

The need for robust, straightforward, rapid, and cost-effective methods to assess thiol redox status stands as a pressing concern within modern analytical science. Although numerous approaches have been proposed to address this concern, their seamless integration into clinical practice is hindered by persistent challenges associated with sample preparation, skill requirements for personnel, and equipment expenses. The currently available glutathione determination kits on the market do not instill complete confidence in researchers. For example, in [33], the authors highlight the inappropriate selection of a masking agent, 2-vinylpyridine (2-VP), for glutathione in the Cayman and Arbor Assays kits. The utilization of 2-VP has been shown to lead to an overestimation of detectable GSSG content and significant errors in quantifying the GSH/GSSG ratio. The Sigma-Aldrich Fluorometric Thiol Assay Kit is not suitably optimized for handling proteins like HSA. Disregarding HSA in samples is impermissible, particularly in the study of pathologies such as renal failure, diabetes mellitus, age-related cataracts, and liver dysfunction, among others [25]. Consequently, advancements in scientific approaches for evaluating thiol redox status and oxidative stress are indispensable for enhancing clinical laboratory diagnostics. For convenience’s sake, a summary of all methods mentioned in this review is provided in Figure 3.

### 3.2. Sample Preparation of Biological Fluids for Thiol Redox-Status Assessment at Their Natural Levels

The process of sample preparation holds critical importance, as it profoundly influences the accurate determination of thiols in biological fluids. Given the high susceptibility of thiols to oxidation within biological samples, safeguarding the sulfhydryl group becomes imperative. Any unintentional decrease in the concentration of RSH during storage or improper sample preparation could cause an increase in RSSR. This aspect assumes fundamental significance when evaluating the redox status of systems characterized by a relatively high ratio of reduced to oxidized forms of thiols. In the last decade and a half, a range of compounds have been employed to protect the sulfhydryl group of diverse thiols, including *N*-ethylmaleimide (NEM) [90,91,92,93], *N*-phenylmaleimide (NPM) [94,95], iodoacetamide (IAM) [93,96], sodium iodoacetate [97], methylmethantiosulfonate (MMTS) [93], 2-VP [90], 2-chloro-1-methylpidinium tetrafluoroborate (CMLT) [98], 2-chloro-1-methylquinoline tetrafluoroborate (CMQT) [99], 4-(5-methanesulfonyl-[1,2,3,4]tetrazol-1-yl)phenol (MSTP) [100], and numerous others.

The pursuit of new and effective masking agents stems from several compelling reasons. A key criterion for these agents is their swift interaction with thiols at physiologically relevant pH levels. This is crucial since, as previously mentioned, acidification can prematurely induce the oxidation of the analyte. Regrettably, this crucial requirement is not consistently met, as published elsewhere [33,101,102]. Knowledge regarding the specificity of masking agents is continually evolving. Recent research has demonstrated that widely employed agents like IAM, NEM, and MMTS can also concurrently interact with sulfene groups [99]. The selection of a reagent for sample preparation is guided not only by its reactivity and specificity but also by its accessibility, the nature of the analyte (whether all thiols or specific ones are being determined), and the characteristics of the object under analysis. In certain studies, masking is coupled with additional derivatization processes, such as the introduction of a fluorescent label. While a universally accepted protocol for the sample preparation of biological specimens for thiol determination is yet to be established, it is imperative to account for the limited window of sample availability post-collection [33].

Diverse approaches to the sample preparation procedure led to notable discrepancies in measured concentrations of RSH and RSSR, as well as in the calculated molar ratios of RSH/RSSR. These variations are observed across different laboratories. With whole-blood samples analyzed via high-performance liquid chromatography–tandem mass spectrometry (HPLC-MS/MS) with ostensibly similar sample preparation protocols, discrepancies persist. This is evident from the data presented in Table 2, which highlights methods for thiol determination in biological fluids. Common approaches for blood samples encompass centrifugation for serums and storage at −80 °C until analysis [103]. The therapeutic target of clinical relevance is represented by the thiol/disulfide ratio in extracellular fluids [104].

In two papers, refs. [96,99], the total content of biothiols in the object was measured, including disulfides with proteins. Also, in [105], measured concentrations of biothiols are presented as mol/gram; for conversion, blood density is taken as 1.05 g/mL. Its activity is primarily due to a terminal sulfhydryl group (Cys34) sensitive to free-radical oxidation. Nanoflow liquid chromatography and high-resolution tandem mass spectrometry (nLC–HR-MS/MS) were used for terminal sulfhydryl group (Cys34) determination [106].

**Table 2:** Thiol content in biological fluids measured by chromatography and spectrophotometry (authors’ data from the original papers were adjusted by rounding off the numbers to show the appropriate number of significant digits).

Object	Analyte(s)	Derivatizing Agent	Thiol Content, μM	Linear Range, μM	LOD, μM	LOQ, μM	Reference
**HPLC-UV**							
Blood plasma	Hcys (total content)	CMQT	5.8	1.0–40	0.15	0.4	[99]
	Cys (total content)		230	1.0–40	0.08	0.2	
	GSH (total content)		5.4	20–300	0.15	0.4	
	HSA (total content)		0.70	3.1–37.5	0.15	0.5	
Saliva	Hcys	CMLT	0.20–2.10	0.1–20	n/m ^1^	0.05	[98]
	Cys		0.60–10.40	0.1–20		0.05	
	GSH		1.70–17.40	5–300		0.08	
**HPLC-FL**							
Whole blood	GSH (total content)	1,3,5,7-Tetramethyl-8-bromomethyl-difluoroboradiaza-s-indacene	15	0.001–0.2	2 × 10^−4^		[107]
	Cys		75	0.005–0.8	8 × 10^−4^		
	*N*-acetylcysteine		15	0.001–0.2	2 × 10^−4^		
	Hcys		30	0.002–0.2	3 × 10^−4^		
**HPLC-MS/MS**							
Whole blood	GSH	NEM	900 ± 140	25–500	0.4	1.5	[91]
	GSSG		1.2 ± 0.4	0.1–16	0.1	0.1	
Whole blood	GSH	NEM	1110 ± 20				[105]
	GSSG		1.6 ± 0.5				
Blood plasma	Hcys (total content)	IAM+IPCF (isopropyl chloroformate)	10 ± 4	0.05–100	0.5 × 10^−3^	10 × 10^−3^	[96]
	Cys (total content)		200 ± 40		0.5 × 10^−3^	20 × 10^−3^	
	GSH (total content)		5 ± 2		0.5 × 10^−3^	10 × 10^−3^	
**GC-MS**							
Saliva	Cys	MSTFA-TMCS (Silylation Reagent)	n/m ^1^	1–20	0.1	n/m ^1^	[108]
	Homocysteine (Hcys)				0.1		
	Homocysteine thiolactone (HTL)				0.05		
**Spectrophotometry**							
Blood plasma	Low-molecular-weight thiols in reduced form (RSH)	DTNB (5,5′-dithiobis-(2-nitrobenzoic acid) + CH_2_O	n/m ^1^	up to 4000	2.8	n/m ^1^	[109]
	Low-molecular-weight thiols in oxidized form (RSSR’)		16.0 ± 0.1	up to 2000	n/m^1^		

^1^ n/m is not mentioned.

A recent review published in mid-2024 provides a thorough understanding of sample preparation techniques used for analyzing pharmaceuticals in complicated matrices [110]. It mentions that CDs have merits over traditional carbon-based nanomaterials. Also, the fundamental tenets of magnetic molecularly imprinted polymers (MIPs) and their applications and the challenges of analysis in the magnetic micro-solid-phase extraction (MSPE) process have been illustrated [110].

### 3.3. UV/Vis Determination of Thiol Redox Status in Biological Fluids

**Figure 4:**
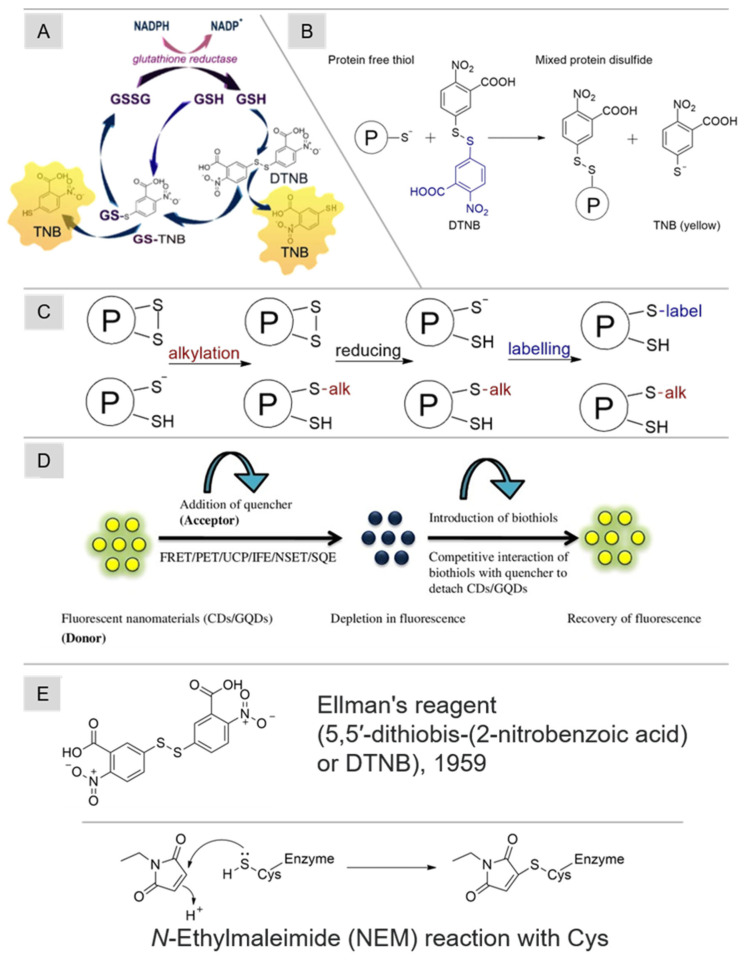
(**A**) The scheme of reactions for the determination of thiols using Ellman’s reagent [33], (**B**) the determination of free reduced thiols with Ellman’s reagent [27], (**C**) a general approach to thiol speciation by monitoring the reversible oxidation of protein thiols based on tags [27], (**D**) the mechanism of thiol detection using fluorescent carbon nanosensors [29], and (**E**) the chemical formulae of DNTB and NEM, as well as the reaction scheme of NEM with Cys. Adapted with permission from [27,29,33]. Copyright 2024, Elsevier.

The traditional method for quantifying reduced thiol levels and evaluating thiol-related oxidative stress involves colorimetric analysis utilizing the Ellman reagent (1959) (5,5′-dithiobis-(2-nitrobenzoic acid), DTNB). To minimize the occurrence of Cys oxidation and the subsequent formation of disulfide bonds, it is essential to subject all solutions to a thorough purging process with either argon or nitrogen gas before utilization [111]. In this technique, Ellman’s reagent engages with thiols in the sample through a disulfide exchange reaction, resulting in a mixed disulfide and 5-thio-2-nitrobenzoic acid (TNB), which exhibits a yellow hue [112]. A scheme of thiol determination by this method is shown in Figure 4B. The spectrophotometric approach measures the absorption intensity of TNB at 412 nm. Ellman’s reagent is versatile, allowing measurements in both model systems and biological samples. For determining low-molecular-weight thiol concentrations, the initial precipitation of protein constituents in the sample is achieved with trichloroacetic acid. The assessment of *N*-acetyl-L-cysteine ethyl ester (NACET) using Flow Injection Analysis (FIA) with subsequent spectrophotometric detection employing various thiol-sensitive ligands has also been presented [113].

A modification of the Ellman reagent technique was introduced and widely employed for assessing both the reduced and oxidized forms of glutathione, as outlined in [114,115]. This method incorporated a cyclic reaction model (Figure 4A) for the reduction of the glutathione adduct of TNB (GS-TNB) and GSSG disulfides, facilitated by the following:

(1) Glutathione reductase (GR);
(2) Nicotinamide adenine dinucleotide phosphate (NADPH).

Consequently, the combined content of GSH and GSSG could be determined. The further addition of NEM or 2-VP permitted the specific determination of GSSG content. While this approach was validated primarily on plasma and commercial kits for GSH determination are based on it, the current literature highlights issues related to NEM (causing side inhibition of GR action) or 2-VP (slow interaction with thiols). Notably, owing to thiols’ heightened sensitivity to oxidation, which is pH-dependent and associated with their p*K*_a_, the precise evaluation of thiol oxidative stress using Ellman’s reagent may be challenging [116].

This highlights off-laboratory analysis and offers promising prospects for practical applications in thiol detection [117]. A visual approach based on thiol–ene click chemistry and the capillary action principle [118] for the molecular recognition of target analyte molecules containing thiol groups was developed. This characteristic has practical implications in point-of-care tests, specifically in scenarios where naked-eye differentiation of results is feasible. For GSH, LOD was estimated at 2 μM with high selectivity, and the measured intra-assay coefficients of variation were lower than 7%.

Current research directions deal with NP applications and the development of a simple, user-friendly, and low-cost photometry procedure for thiol determination based on their inhibition of AuNPs, with quantification through UV-vis spectroscopy (photometry) and RGB-based colorimetric analysis. This procedure exhibits effectiveness in measuring the concentrations of glutathione in red blood cells and cysteine in plasma, with recoveries of up to 97%, low detection limits (up to 1.0 μM), and reliable selectivity [119]. The addition of biothiols to the AuBr_4_^−^–CTAB complex results in the initial quenching of coloration and induces a subsequent decrease in absorbance, as measured by a microtiter plate photometer. Biothiols were determined in urine and blood plasma samples; the procedure yields satisfactory recoveries (up to 115% with standard addition) and demonstrates good reproducibility (RSD < 10%) [120].

### 3.4. Luminescent Sensors for Thiol Redox-Status Determination

The increased interest of researchers is related to the development of luminescent sensors and actuators for biothiols. These sensors offer a straightforward design, relatively high sensitivity values ranging from 20 nM to 100 µM, rapid analysis, and easy accessibility, positioning them as burgeoning tools for the real-world assessment of redox status. Carbon-based fluorescent materials, including carbon dots and graphene quantum dots, can serve as sensors [29,121,122,123,124]. Their strengths encompass high water solubility, photoluminescence stability, and good biocompatibility. Using carbon nanomaterials poses challenges related to potential biological toxicity, especially when employing a turn-off approach to capture the analytical signal [125]. Various intricacies are tied to sample preparation. On the one hand, samples could potentially be analyzed immediately after collection, bypassing the need for derivatization. On the other hand, the production of sensors that maintain the necessary analytical characteristics of untreated biological matrices is a notably labor-intensive task [126]. The mechanism of thiol detection by such materials is shown in Figure 4D.

There are data on the oxidative modification of thiol-containing proteins and amino acids by interaction with Ce(IV). A straightforward, swift, and highly sensitive method for determining the total quantity of free thiol groups in biological samples was introduced based on the oxidative capability of Ce(IV) [127]. The investigative protocol involved utilizing Ce(IV) as a probe: thiol oxidation results in a Ce(III) and thiol/disulfide complex, exhibiting fluorescence at 352 nm [127,128]. In a study focusing on the interaction between metallothioneins (a family of low-molecular-weight proteins rich in cysteine), cysteine, and CeO_2_ nanoparticles, researchers explored the potential impact of the Ce(IV)/Ce(III) redox system on the thiol groups of these biomolecules using diverse biophysical approaches [129]. Notably, fluorescence analysis highlighted the formation of stable disulfide bridge/Ce(III) complexes, explaining the interaction between Ce(IV) and thiol groups.

Chemosensors based on metal complexes with organic ligands are also presented [130,131,132,133]. Most of these sensors have been tested only on model mixtures of biothiols and cell samples. Other examples of optical sensors for determining biothiols are presented in Table 3.

It is important to highlight that the fluorescence approach is highly helpful beyond just thiol detection. The DCFH assay, also known as a 2′,7′-Dichlorodihydrofluorescein assay, has been used for over 50 years to detect ROS [134]. This is a widely employed method that measures the production of intracellular ROS. The compound undergoes a reaction with free radicals, resulting in the formation of a fluorescent product. At present, there is a significant body of literature focused on optimizing traditional plate-reader-based DCFH assay protocols to adhere to the technical-specification standard protocol (flow cytometry) regarding improving reliability and sensitivity [135].

**Table 3:** Determination of biothiols using fluorescent sensors.

Type of Luminescence	Optical Sensor	Object	Analyte(s)	LOD, μM	Linear Range, μM	Reference
Fluorescence	Au(FR 730) (nanoparticles)	Blood plasma	Cys	0.01	2.5 × 10^−2^–4.0	[136]
	DMAT-π-CAP	Urine	HSA	0.01	0.01–10	[137]
	Hg^2+^_2_(murexide)_2_	Blood serum	GSH	0.01	0.1–40	[138]
			Cys	0.02	0.5–30	
			Hcys	0.04	0.5–50	
Chemiluminescence	CdTe (quantum dots)	Blood serum	GSH	2 × 10^−3^	(2–650) × 10^−3^	[139]
	System of TGA-CdTe (quantum dots), Au(NaBH_4_), Au(citrate), Ag(citrate) (nanoparticles)	Blood plasma	GSH		5–800	[140]
			GSSG		5–800	
			Cys		25–100	

### 3.5. Chemiluminescent Methods for Studying Thiol Oxidative Stress

A promising direction is the study of thiol pools by chemiluminescent methods. The phenomenon of chemiluminescence is used for detection in chromatographic analysis and flow-injection systems [141] and underlies the process of chemiluminometry. Chemiluminescence (CL) refers to light emission resulting from a chemical reaction. During a chemiluminescent reaction, one or more products are formed in an electronically excited state, which emits light photons upon relaxation.

Presently, considerable attention is focused on nanoparticle-enhanced luminol-dependent CL. Integrating nanoparticles into chemiluminescent systems significantly enhances the light signal and leads to a notable increase in analysis sensitivity [142,143]. Nonetheless, a remaining considerable challenge in chemiluminescence analysis is the simultaneous and selective determination of multiple analytes. To address this issue, the diverse range of nanomaterials developed to date, combined with chemometric tools, offers a promising solution and substantially expands the potential applications of luminol-dependent CL. A noteworthy example is [140], where the authors proposed a method for the selective determination of Cys, GSH, and GSSG using an array of nano-sized chemiluminescent probe sensors. The chemiluminescence intensity displayed varying degrees of change upon interaction with biothiols, resulting in unique chemiluminescence response patterns. Four luminol-based CL systems like luminol and H_2_O_2_ with quantum dots TGA-CdTe (TGA, thioglycolic acid), luminol–H_2_O_2_ with citrate-capped silver nanoparticles (Cit-AgNP) (Cit, citrate), luminol–AgNO_3_ with citrate-capped gold nanoparticles (Cit-AuNP), and luminol–AgNO_3_ with grafted borohydride-capped gold nanoparticles (BH_4_-AuNP) exhibited distinct response patterns for Cys, GSH, and GSSG. These specific CL response patterns were treated as “fingerprints”, relying on the values of the CL increment (Δ*I*_CL_), and subsequently identified using chemometric methods, including linear discriminant analysis (LDA) and hierarchical cluster analysis (HCA). The developed array demonstrated the capability to discriminate cysteine, glutathione, and glutathione disulfide in the human blood plasma matrix across broad concentration ranges (5–800 μM for GSH and GSSG and 25–100 μM for Cys). The integration of nanohybrids (specifically Au-activated luminol) can substantially enhance the selectivity of chemiluminescence speciation analysis, leading to the reliable and accurate determination of cysteine instead of homocysteine and glutathione [144].

Chromatographic separation with chemiluminescence detection is the most sensitive and prominent method for separating and quantifying thiols. Chemiluminescence detection is the preferred method for biological samples [145,146]. A key advantage of using CL to detect biological sample components is high sensitivity and selectivity. This is due to the uniqueness and rarity of the CL phenomenon itself and to the competent selection of CL activators or derivatizing agents.

A post-column reaction with a chemiluminescent reagent is usually performed to determine thiols using CL, which minimizes the sample preparation stage. For this purpose, thiols must be separated in their native form as they pass through the column. The choice of the mobile phase is also essential: its requirement provides a satisfactory separation and is a favorable medium for the chemiluminescence reaction. These requirements are satisfied by dilute trifluoroacetic acid mixed with methanol [28].

Quinone compounds have shown significant potential as reagents for chemiluminescence detection. Quinones are highly reactive and nucleophilic, enabling them to form stable and reactive complexes with various analytes. In the study referenced [147], quinones were utilized to create stable adducts with aminothiols in the presence of dithiothreitol, which triggered the generation of ROS. Subsequently, ROS-induced chemiluminescence was measured to determine aminothiol concentrations in blood plasma samples from healthy individuals and patients diagnosed with rheumatoid arthritis and diabetes mellitus. In this case, the linear ranges for GSH, *N*-acetylcysteine, Hcys, and Cys were 2.5–500, 5–500, 10–1500, and 20–2000 nM, and the limits of detection (determined as 3× signal-to-noise ratio) were 3.8, 4.2, 8, and 16 (fmol per injection), respectively.

In [148], the chemiluminescence detection of thiols (Cys, GSH, Hcys, *N*-acetylcysteine) and their disulfides (cystine, GSSG, homocystine) in a single chromatographic separation using a Mn(IV) reagent was described. The technique was tested on whole-blood samples, where GSH and GSSG levels were determined. The authors also evaluated the GSH/GSSG ratio for 12 healthy donors aged 21 to 31 years, and the values ranged from 5 to 9, with a mean value of 6.6. However, there are concerns that the manganese(IV) reagent can oxidize methanol and acetonitrile in the mobile phase with the release of intense light, which degrades the sensitivity of the analysis [149].

The use of triangular gold nanoparticles as a post-column reagent under luminol-dependent CL conditions is shown in [150]. Thus, blood plasma and urine were analyzed, where Cys, GSH, Hcys, cysteinyl–glycine (Cys-Gly), and glutamyl–cysteine (Glu-Cys) levels were determined. For each of the matrices, the method was characterized by a wide linear range and low detection limits (0.02 to 0.1 pM). However, the previously mentioned system is limited in detecting disulfides, necessitating a separate disulfide reduction step before the second chromatography run. To address this issue, [149] proposed an improved approach by incorporating the online reduction of disulfides. The modification involved treating the quartz column with crystals of Zn(II)-TCEP complexes, which were synthesized using tris(2-carboxyethyl)phosphine (TCEP) and Zn(ClO_4_)_2_ in a mixed aqueous solution of NH_3_ and methanol. The Zn(II)-TCEP complexes could efficiently reduce disulfide bonds at an acidic pH of 3.0, compatible with the mobile phase used in the HPLC separation of thiols and disulfides. The use of Zn(II) is highly justified due to its high reduction potential [151], E(Zn(II)/Zn(0)) = −0.76 V, which cannot, e.g., cause solvent oxidation. To the contrary, in [152], it is established that Mn(IV) may oxidize organic solvents (e.g., methanol and acetonitrile) in the mobile phase to emit intense light, which deteriorates the sensitivity of the analysis, increasing the black signal. Also, such behavior was shown for metal–organic frameworks with Ce(IV) that have a strong oxidizing ability toward dyes [153]. This can be explained by checking the reduction potential [151] in acidic media: for the Ce(IV)/Ce(III) pair, it is 1.72 V, and for Mn(IV)/Mn(II), 1.22 V.

The native thiol and disulfide peaks were separated and also underwent reduction inside the column. Like the previous setup, the CL system for thiol detection included luminol, H_2_O_2_, and triangular gold nanoparticles. With this improved method, the limits of detection achieved for cystine, Cys, Cys-Gly, homocystine, Hcys, GSH, and Glu-Cys in human urine and plasma samples ranged from 10 to 25 nM, and the linear range on the logarithmic graph extended up to three orders of magnitude. These values are sufficient for working with two-hundred-times-diluted biological fluids.

Conducting a pre-column reaction is less common. In [154], Hcys was determined in the blood plasma of healthy donors. Hcys content ranging from 0.025 to 5 μM caused a dose-dependent change in CL intensity. The detection limit was 8 nM. Here, separation by capillary electrophoresis (CE) was preceded by sample treatment with *N*-(4-aminobutyl)-*N*-ethylisoluminol (ABEI).

### 3.6. Other Spectroscopic Procedures (FTIR and Raman) for Thiol Redox-Status Assessment

Chemical signal amplification is the most prominent part of the analytical procedure for various methods [155]. The approach can increase the sensitivity of the analysis at a minimal instrument cost and with a short timeline. Several techniques involving signal amplification include the following:

- Polymerase chain reaction [156];
- Catalysis [157];
- Complexes with known and specified stoichiometry, like hetero-poly complexes based on multivalency [158].

However, there is no evidence for the use of chemical signal amplification or “chemical amplification” [159] to determine biothiols. Another approach deals with the physical amplification of the signal by surface-enhanced Raman scattering (SERS), which may have possibilities for biomolecule analysis [160]. The parameters of the thiol (–SH)/disulfide bond (–S–S–) redox process were measured by Fourier-transform Raman spectroscopy (FT–Raman) and terahertz time-domain spectroscopy (THz–TDS) in [161]. The dynamic response of the cysteine/cystine experimental spectrum and characteristic peaks were analyzed by solid-state density functional theory, which can quantitatively analyze the ratio of cysteine/cystine. Improved drug safety through accurate enantiomer identification is vital. The study proposed a simple, sensitive method combining principal component analysis with terahertz spectroscopy to distinguish between chiral L- and D-cystine molecules [162,163]. SERS analysis with a silver colloid substrate to quantify biothiols in small-volume whole-blood (umbilical cord) samples. The study established a statistically significant correlation between SERS signals and thiol concentrations measured through a chromatographic reference method for accuracy estimation in umbilical cord WB samples. It developed a Partial Least Squares (PLS) regression model covering GSH concentrations from 10 to 2200 μM. The approach shows promise for clinical applications, particularly neonatology, offering a reliable and rapid tool for directly determining thiols and enhancing healthcare outcomes [164].

The authors of [165] explored the application of FTIR spectroscopy for the simultaneous quantitative analysis of 38 components in blood serum, demonstrating its potential as a reagent-free diagnostic tool. The study was limited by using model systems with constant component ratios. It also faced challenges from the complexity of real blood samples, potentially affecting accuracy. Further validation with clinical samples is necessary to confirm the method’s reliability and robustness. The study underscores the precision of this approach, achieving measurement errors below 0.1%. The purpose is to validate FTIR spectroscopy as an efficient alternative for routine blood serum analysis, effectively meeting the demand for cost-effective and non-invasive diagnostic methods in healthcare settings.

### 3.7. Chromatographic Determination of Thiol Redox Status in Biological Fluids

Despite the broad research carried out on the GSH/GSSG system and the recent progress in measurement techniques, the lack of agreement or standardization approaches among assays remains a substantial issue. This is intensified by the possible influence of artifacts arising from improper sample handling and sample preparations on the determined GSSG concentration. Consequently, there is an urgent requirement for integrative approaches that address these errors and include other thiol-containing biomolecules [166].

One widely adopted method for analyzing alterations in specific thiol pools within plasma is high-performance liquid chromatography with fluorometric detection (HPLC–FL). This chromatographic separation can incorporate pre- or post-column reaction labeling of thiols. Reagents capable of forming fluorescence-capable conjugates with thiols include monobromo(trimethylammonium)bimane (qBBr), monobromobiman (mBrB), 7-fluorobenzo-2-oxa-1,3-diazole-4-sulfonate (SBD-F) [167], 4-(aminosulfonyl)-7-fluoro-2,1,3-benzoxadiazole (ABD-F) [112], etc. These compounds offer heightened sensitivity compared to Ellman’s reagent. When coupled with sodium dodecyl sulfate–polyacrylamide gel electrophoresis (SDS-PAGE), they can also detect modified proteins. A general approach to monitoring the reversible oxidation of protein thiols based on tags is shown schematically in Figure 4C. Here, the analytical characteristics of thiol detection are significantly affected by the fluorescent label used. In [168], a comparison of the linearity ranges and detection limits achieved by different HPLC-FL techniques was presented. Thus, the best sensitivity was achieved by MIPBO (5-methyl-(2-(*m*-iodoacetylaminophenyl)benzoxazole) (detection limit, 3.5–15 fM for Cys and GSH) and ABD-F (*LOD* = 2–10 pM for Cys, Hcys, and GSH). The most convenient for work (in the context of redox-status estimation) in the linear range is obtained using monobromobiman 0.5–750 µM for Cys, Hcys, and GSH. Chromatographic analysis of thiols in biological fluids is not confined solely to fluorometric detection. While fluorescence offers convenience, it is not without challenges. Certain fluorescent tags produce numerous fluorescent byproducts, necessitate prolonged time and elevated temperatures during derivatization [94], and are also prone to photodegradation.

Chromatography–mass spectrometry has emerged as a burgeoning alternative method. The determination of thiols in biological samples has extended to include gas chromatography–mass spectrometry methods [102,169,170], high-performance liquid chromatography–mass spectrometry (HPLC-MS), and HPLC-MS/MS [91,94,95,96,167,171,172,173,174]. Within the realm of HPLC, the separation of biological fluid sample components is achieved in reverse-phase mode using C18-type columns or hydrophilic interaction liquid chromatography.

Currently, robust, versatile, and advanced ultra-HPLC systems with heated electrospray ionization (ESI) sources have been developed. These techniques enable the concurrent analysis of the thiol redox metabolome, encompassing total and free thiols, disulfides, and sulfide, within intricate biological samples like human blood, saliva, and urine [175]. Given the vital function of GSH, comprehending its biological significance requires a thorough understanding of its synthesis and metabolism, as outlined in the subsequent text. The latest studies are examined and critically discussed regarding the potential for the exogenous regulation of GSH levels through food supplementation. A section of the review is also devoted to the analysis of GSH through non-separation and separation techniques, particularly by HPLC [176].

Recently, implementing sample preparation and derivatization methods has significantly improved the sensitivity and accuracy of detection. The focus in the future will be on the development of detection methods that are eco-friendly, cost-effective, and more sensitive. The combination of chromatography and MS provides exceptional sensitivity and specificity in thiol analysis within biofluids [32].

In the past decade, the preferred choice for thiol analysis between targeted and non-targeted analytical procedures [177] has consistently been the first approach, primarily due to its greater suitability and ease of quantification. Using HPLC-MS/MS enables a thorough investigation of thiol levels in cultured cells, providing valuable insights into their intracellular redox status [178].

The ability of chromatography–mass spectrometry to concurrently determine the content of both reduced and oxidized forms of multiple thiols is a significant aspect in assessing redox status. In [96,173], the quantification of thiols and their disulfides in blood plasma has been achieved in the picomole range, with ultra-trace levels ranging from 0.5 nM to 1 fM.

However, low LOD values are not a requirement for assessing thiol redox status since thiol concentrations under study are usually significantly higher. Notably, in [96], the authors not only determined the content of thiol components in blood plasma but also calculated the redox-couple potential values for each analyte. As this parameter hinges on the thiol/disulfide ratio of compound modifications in the medium, it holds potential for clinical diagnostic purposes. Table 2 presents all information on thiol analysis using chromatography.

### 3.8. Electroanalytical Methods for the Assay of Low-Molecular-Weight Thiols and Albumin

Thiol substances play an active and crucial role in redox processes within living systems, as emphasized multiple times in this review. Electrochemical methods are effective in detecting and quantifying thiol antioxidants based on voltammetry and/or amperometry. The most prominent benefit concerning electroanalysis may be the analysis of colored media without sample preparation. Several limitations of electrochemical techniques in analyzing biological fluids relate to a potential lack of selectivity [179].

Speaking of selectivity, glucometers are the most prominent electrochemical devices just for whole-blood analysis. This analysis is conducted for an undiluted, colored sample, and this is highly selective. However, the key factor in this determination of glucose is the coupling of an enzymatic reaction with an electrochemical one [180]. The disadvantages of this type of sensor are the stability of enzymes, their cost, and their demanding storage conditions [181].

When examining oxidative stress in electrochemical studies conducted over the past decade, glutathione has been the focus of investigation most frequently [182], as well as the assessment of cysteine and homocysteine [183]. The approaches and trends outlined for studying glutathione also apply extensively to other low-molecular-weight thiols, such as L-cysteine and homocysteine. The commonly reported methods in the relevant literature are cyclic voltammetry, linear sweep voltammetry, differential pulse voltammetry, or square-wave voltammetry. Cyclic voltammetry is frequently utilized to examine the thermodynamics and kinetics of redox processes, enabling the formulation of analytical experimental conditions. The selection of electrolyte pH, potential range, and scanning speed is guided by the voltammograms obtained in the cyclic voltammetry mode [184]. For the quantitative determination of low-molecular-weight thiols, linear sweep voltammetry, differential pulse voltammetry, and square-pulse voltammetry are generally preferred due to their superior sensitivity and selectivity compared to cyclic voltammetry [30,185]. When faced with situations that involve (1) the simultaneous determination of multiple analytes with closely matched redox potentials or (2) the accurate estimation of thiol content in the presence of compounds possessing lower redox potentials, the selectivity of these methods may be insufficient. This scenario is frequently encountered when working with biological fluids.

An important review (a part of a themed collection) concerning electrochemical assays of reduced (GSH) and oxidized glutathione (GSSG) presented a map of various detection routes [186], which included (a) indirect methods (solely for GSH through byproduct monitoring); (b) electrochemical oxidation and electrochemical reduction (both GSH and GSSG through mediated and direct analysis); and (c) combinations with separation techniques.

As mentioned above, one of the possible solutions is to combine electrochemical detection (usually using an amperometric or coulometric detector) with pre-separation by HPLC or CE. Thus, the separation of sample components allows us to turn to simpler electrochemical methods and does not require the development of new variants of working electrodes. The electroactivity of S-moieties allows for the determination of cystine, cysteine, homocysteine, methionine, glutathione, glutathione disulfide (GSSG), ascorbic acid, and tyrosine in rat plasma [187] or human cell lines [188] using HPLC with amperometric detection. Since disulfides (GSSH, Cyss) are the oxidized species of sulfides, one of the challenges of HPLC coupled with amperometric (or coulometric) detection was to avoid preliminary sample preparation, mainly the reduction of disulfides. Chromatography coupled with pulsed amperometric detection or multiple electrochemical cells with different applied potentials (eight cells with applied potentials of 0.400–0.850 V vs. Pd were used [187]) allowed for the simultaneous determination of several aminothiols and disulfides, as well as a much-simplified preparation of plasma samples, which only required the transfer of 100 or 200 μL of plasma to a new tube with the same cold volume of 2% protocatechuic acid/0.2 M boric acid [187]. However, the chromatographic conditions employed in this study may not be optimal, especially if the aim is to implement the method in routine practice. The mobile phases in the mentioned study include the ion-pair modifier 1-octanesulfonic acid, which typically requires a significant column conditioning time before analysis [189]. The analysis duration in this study was 40 min, which can be considered relatively long. Also, there are questions about the stability of the samples while in the autosampler (sample storage conditions, according to the authors, are −80 °C). The same storage conditions are required for lysed cancer cell samples [188]. The analysis duration has been extensively addressed in many studies on this subject. Despite this, the authors achieved satisfactory reproducibility, as evidenced by repeated regression analyses of the standards or samples, yielding *r*-values ranging from 0.980 to 0.995. The measured concentrations were in the nano- and micromolar ranges, with specific values provided for cysteine, cystine, glutathione, and glutathione disulfide after treating rats with kainic acid or pilocarpine to induce epilepticus status.

Therefore, thiol/disulfide ratios may serve as blood biomarkers of epileptic seizure progression and treatment response in epilepsy patients [187]. In [188], the GSH/GSSG ratio was used as an oxidative stress indicator and was found to be different in an immortalized normal cell line and malignant cell lines, confirming important metabolic differences.

Thiol analysis requires high anodic potentials, because the electrochemical signal corresponds to the oxidation of the analyzed thiol compounds. Therefore, selecting the electrode material is important for electrochemical detection. For carbon electrodes, the working potential window does not allow the oxidation of GSH or GSSG in aqueous media. The recent development of diamond electrodes made of boron-doped diamond with wide electrochemical windows opened possibilities for the simultaneous oxidation of thiols and disulfides in an amperometric regime under a constant potential within the same analysis [188]. Other examples of combining HPLC or CE with electrochemical methods can be found in [190,191,192].

To improve the selectivity and background stability, detectors may be equipped with dual (or more) electrochemical cells and several electrodes. One of the electrodes is set at a lower potential than the second one, helping to eliminate interfering compounds with lower oxidation potentials than the thiols of interest [190,192]. The on-chip CE method with multiple microelectrodes decreases the detection time of Cys and HCys by up to 2 min and miniaturizes the setup to a microchip device [191].

Also, a comparison of optical and electrochemical methods for in vivo analysis of GSH in animal tissues has been shown [193]. Among the benefits of HPLC coupled with electrochemical detection, simple sample preparation and accuracy were emphasized. However, commonly employed electroanalytical strategies are hybrid methods that involve separating sample components using HPLC or CE in combination with electrochemical detection. Solely electrochemical devices and sensors for the direct electrochemical detection of thiols in complex matrices also deserve attention.

To address insufficient selectivity in electrochemical methods, researchers have explored modifying existing working electrodes or developing new electrode materials, which will be further discussed [194].

Here, in this review, we group the information by modified electrodes and unmodified electrodes, not by the electroanalytical methods themselves. The registration mechanism is considered, which can be one of the following:

- The direct registration of the analytical oxidation or reduction signal of thiol compounds, with or without pre-separation;
- Registration with modifiers based on one of the following processes:
  (a) A catalytic mechanism (enzymes and nanohybrids of nanoparticles);
  (b) Specific interactions, e.g., MIPs;
  (c) Mediator-based variants (that is, the signal is not recorded directly but by a redox indicator).

A promising direction for analyzing the thiol content in biological fluids outside of laboratory settings is the modification of screen-printed electrodes. In comparison to noble-metal electrodes, carbon materials are more available, cheaper, and biocompatible. Modified carbon electrodes, including glassy carbon and carbon paste, are commonly employed as working electrodes for determining low-molecular-weight thiol antioxidants in biological fluids [195,196,197,198,199]. The development of new electrode materials or the modification of existing electrodes changes the oxidation/reduction potentials and enhances their selectivity or improves simultaneous electrochemical speciation for the redox profiling of oxidative stress. For example, the modification of a glassy carbon electrode with a nanocomposite based on cobalt phthalocyanine resulted in electrocatalytic activity for GSH oxidation (at *E* = −0.10 V vs. Ag/AgCl) and GSSG reduction (at −0.72 V), providing the simultaneous detection of both in biological samples without any pretreatment [198]. The modifications applied to these electrodes aim to increase their surface area, provide specific binding sites, or create a barrier that reduces interference from other compounds. These modifications enhance the selectivity and sensitivity of thiol determination. Carbon-based electrodes are favored over noble-metal electrodes due to the ease of passivation of the metal surface by forming either metal oxides under specific electrochemical conditions or covalent metal–sulfur bonds. In some cases, noble-metal modifiers, such as gold nanoparticles (AuNPs), enhance the performance of carbon-based electrodes [197,200]. Noble metals can serve as catalysts for specific nitrosothiol reactions in thiol determination [201]. In this study, a novel electrochemical technique rather than an electrode was developed. Nitrosothiol homolytically cleaved by gold nanoparticles released NO, which was measured with a NO-selective electrode. These studies show the successful utilization of carbon electrodes modified with nanoparticles or carbon nanotubes for the sensitive determination of thiol compounds in various complex biological fluids.

An electrochemical assay for homocysteine using carbon screen-printed electrodes with cytochrome-c-anchored gold nanoparticles allowed its detection with an LOD level of 0.3 µM [202]. The electrocatalytic action of cytochrome’s Fe^3+/^Fe^2+^ active center led to the lower oxidation potential of HCys and improved the kinetics of the process. However, a real sample analysis still required human plasma reduction with NaBH_4_.

To date, low-molecular-weight thiol electrochemical assays have been actively developed with good selectivity and low detection limits. There are options for enzyme-based biosensors able to detect glutathione in biological fluids and cells using the following components:

(1) Glutathione peroxidase (GSH-Px) in a range from 40 to 2000 µM [186];
(2) Glutathione oxidase (GSH-OX) in a range from 5 to 1000 µM [203];
(3) Nanohybrids with GSH-Px deposited on graphene oxide (GO) and Nafion™, which reduced the LOQ to 0.002 µM, in a range from 0.003 to 370 µM using a differential pulse voltammetric (DPV) method [204].

Other types of biosensors include those based on the following processes:

(4) The inhibition of bilirubin oxidase (or laccase) by GSH in a range from 40 to 2000 µM [205];
(5) Possibilities of glutathione transferases for the construction of biosensors not only for glutathione [206];
(6) A different mechanism of action, demonstrated by an impedimetric immunosensor for low-level human serum albumin detection in biological fluids [200].

Alternative nanoenzymatic approaches to modifying the electrode surface involve immobilizing specific molecules or polymers, which enhances the electrode’s performance in thiol determination [199], reduced glutathione [207], and Cys [208]. Success in the relatively new field of utilizing metal–organic coordination polymers in electrochemical sensing allowed for the detection of GSH based on its electrocatalytic oxidation [207]. The electrocatalytic mechanism is an effective approach to the design of selective thiol electrochemical sensors. Analogs of catechol can act as electron shuttles (mediators) between the electrode and the thiol molecule. For example, quinone/hydroquinone-mediated catalytic oxidation of Cys results in 200 mV lower oxidation potential and higher sensitivity [208]. Particularly noteworthy are the works of Lee and Compton, who successfully determined glutathione and homocysteine when present in synthetic saliva [209], the culture medium of cell tissues, and human blood plasma [210]. The limit of detection for homocysteine and glutathione in synthetic saliva was determined to be 0.9 μM and 2 μM, respectively. In tissue cultures, the LOD was approximately 2 μM for both analytes, while in human plasma, it was ca. 0.8 μM for homocysteine and glutathione. They achieved this using carbon-based screen-printed electrodes composed of multiwalled carbon nanotubes. Their strategy involved two key steps: (a) facilitating the electrooxidation reaction of catechol with thiols through a 1,4-Michael addition reaction [211], resulting in a detectable adduct peak signal, and (b) examining the results at different voltage scan rates [212].

In recent years, the electrochemical determination of albumin has made significant advancements through the development and application of molecularly imprinted sensors [213]. Molecularly imprinted polymers (MIPs) are synthetic receptors for a targeted molecule [214]. Imprinting techniques have already proved their applicability for complex matrices, such as in environmental or food samples, and their high selectivity in drug analysis, where stereoselectivity is necessary for successful drug extraction [110]. These sensors use MIPs as the recognition elements, imparting them with high selectivity for albumin detection. The MIP structure incorporates specific regions known as imprints, which can interact specifically with template molecules or compounds that closely resemble albumin in structure. The selective binding of albumin by MIPs induces changes in various electrochemical properties, such as the impedance, current, or potential, which can be measured and correlated with the albumin concentration. This selective detection mechanism enables the accurate assessment of albumin levels, even at low concentrations [215]. This capability is crucial for detecting trace amounts of albumin in biological fluids. Molecularly imprinted polymers in these sensors exhibit robustness and stability, allowing for their reusable and reliable use [216]. Once synthesized, the MIPs maintain their affinity for albumin over extended periods, ensuring the reproducibility of the sensor response. This characteristic makes molecularly imprinted sensors well suited for the routine analysis of albumin levels. The preference for developing molecularly imprinted sensors, as observed in this case, can be attributed to the structural characteristics of the protein, which enable accurate recognition by the receptor of the electrochemical sensor [217]. The merits of and future plans for molecularly imprinted polymers are provided in [110], showing their capacity, recyclability, and selectivity.

Molecularly imprinted polymers were developed based on bis(2,2′-bithien-5-yl)methane links for human serum albumin analysis in serum [218], and the albumin epitope was used for serum analysis [219] by a molecularly imprinted electrochemical biosensor based on a dual-signal strategy. In these studies, the achieved LOD values ranged from 10 to 100 ng/mL. Notably, ref. [220] demonstrated the most comprehensive linear range, spanning six orders of magnitude (from 10 to 10^5^ ng/L). Hence, MIPs provide an advantageous performance of electrochemical sensors, allowing improved sensor selectivity. Future perspectives of multifunctional sensing or separation platforms include the combination of MIPs with other functional materials, such as redox or fluorescence probes and catalytic, magnetic, or carbon nanoparticles [110]. 

Table 4 presents all of the information on thiol analysis using electrochemical methods.

### 3.9. Machine Learning Tools for Data Processing in Biothiol Analysis

Machine learning (ML) models have become increasingly prevalent in classifying and predicting various biochemical processes. Notably, substantial research has focused on developing these models for assessing and predicting oxidative stress. Also, a review of the application of classical and quantum–mechanical atomistic simulation tools to the investigation of selected relevant issues in thiol redox biochemistry is presented in [221]. By employing supervised machine learning, these technologies effectively automate the evaluation and quantification of oxidative damage in biological samples, extracting valuable insights from experimental data.

This concise review explores the potential applications of neural networks, decision trees, and regression analysis as common strategies in machine learning. It discusses recent highlights and the weaknesses and limitations in biochemistry and related scientific domains [222]. The review explores innovative future approaches, envisioning how ML can contribute to automating oxidative stress measurement and diagnosing diseases linked to oxidative damage by clustering data from different methods. Such advancements in ML might revolutionize how oxidative stress-related conditions are diagnosed and treated [222]. Regarding toxicology research, artificial neural networks (ANNs) hold tremendous potential for toxicity prediction and chemical compound classification based on their effects. Several ANN models have been developed to detect complex chemical–biological interactions and accurately differentiate between damaged and intact cells after toxic exposure.

Convolutional neural networks show skill in recognizing discrete modifications in toxicity-related two-dimensional data patterns. This approach is used for improving precision in the classification of blood-type cells [223], and it has not been used for quantitative analysis to date.

Bayesian neural networks employing weight marginalization may outperform traditional approaches in prediction performance. ANNs are poised to play vital roles in precise and cost-effective biosensors for detecting toxic substances and assessing their biochemical properties [224].

In the medical field, integrating surface-enhanced Raman spectroscopy (SERS) and machine learning (ML) offers great promise. With various medical applications, SERS has shown effectiveness in non-invasive, rapid, and trace analysis. ML, simulating human learning, enhances learning efficiency by structuring existing knowledge. A previous review outlines recent applications of SERS combined with ML, encompassing biological molecule recognition, disease diagnosis, novel immunoassay techniques, and improved semi-quantitative measurements. The review addresses potential opportunities and challenges arising from the amalgamation of SERS and ML in the medical domain [225].

### 3.10. Alternative Indirect Approaches to Determining Thiol Redox Status

The methods described above make it possible to measure the content of the reduced form of thiols (except for chromato-mass spectrometry). The preliminary reduction provides information about the full content of thiol components in the sample, making it possible to estimate thiol redox status indirectly. In parallel, some methods determine the quantity of oxidized forms of thiols.

Nuclear magnetic resonance spectroscopy and mass spectrometry [226] can quantify sulfenic acids in a sample. The previously mentioned colorimetric analysis with Ellman’s reagent also allows the indirect determination of sulfonates since sulfenic acids use externally added thiols (see Figure 4B) [227]. Radioactive labeling of GSH (Tritium, ^3^H) [228] or Cys (^35^S isotope) molecules [229] is widely used to quantify low-molecular-weight and protein thiols and disulfides. The use of non-radioactive biotin or fluorescent tags is also common. This approach allows the selective determination of glutathione disulfides on tissue sections or in homogenates [230]. For this purpose, the target compounds are enzymatically reduced to free thiols and then labeled with a thiol-reactive functionalized reagent. Protein sulfinic and sulfonic acids can be determined using isoelectric-focused gel band shift monitoring [231] or an immunoassay [27]. A Cys-selective reagent that generates a more reactive electrophile upon conjugation could expand the range of nucleophiles usable in current methods for infrared, both NMR ^19^F and ^15^N [232], chemo-selective labeling of albumin [233] and other labeling techniques.

### 3.11. Summary of Applications and Merits of Each Method

By now, a pool of methods and techniques for the determination of thiols in biological fluids has already been formed. Once again, the methods commonly employed include spectroscopy, chromatography, and electrochemistry. Furthermore, these approaches provide the means to identify different sulfur species, not only the total thiol quantity. Some commercially available rapid thiol screening kits have been developed, but they may not be adequately optimized for sulfur-form determination.

Below, Table 5 comprehensively captures information concerning the discussed methods, including their application scope, advantages, selectivity, and sensitivity. The accuracy of thiol measurement and analysis relies heavily on the influence of factors such as sample preparation, so the selectivity of each method will be used.

## 4. Conclusions

### 4.1. Concluding Remarks

According to existing data, the thiol/disulfide ratio plays a crucial role as a regulator of biological processes, making it essential for clinical and laboratory diagnostics to assess the levels of both reduced and oxidized forms of thiols. However, due to the instability of natural antioxidants and their involvement in various simultaneous processes, developing robust and reliable procedures and techniques for their determination is still ongoing for analytical sciences. Modern analytical approaches for determining thiols in biological fluids face challenges such as complex sample preparation, limited access to derivatizing agents or sensor materials, and the need for expensive equipment like gas chromatography–mass spectrometry devices. The simultaneous speciation analysis of reduced and oxidized forms of thiols during a single analysis is relatively rare. Within our review, we make some recommendations for future research that can be segmented into two distinct pathways. For both basic thiol analysis and speciation analysis, accurate and robust quantification can be achieved using spectroscopy and electrochemistry methods. Conversely, for the non-targeted analysis of thiol species, a recently developed chromatography platform can be utilized. This platform enables the simultaneous determination of the thiol redox metabolome, encompassing total and free thiols and their corresponding disulfides, as well as sulfides. The application of this platform extends to complex biological matrices, including human blood.

### 4.2. Forthcoming Advances, Future Perspectives, and Trends

The intricate composition of biological matrices and the potential risks associated with handling biological samples, particularly blood, cannot be underestimated in clinical and laboratory diagnostics. Focusing on these challenges requires analytical methods that are both rapid and compact.

Recent advancements have been made with UHPLC (ultra-high-performance liquid chromatography), HPLC-CL (high-performance liquid chromatography with fluorimetric detection), FIA–CL (Chemiluminometric Flow Injection Analysis), and HPLC–MS (high-performance liquid chromatography–mass spectrometry) variants. In the near future, we can expect ongoing advancements in developing novel derivatizing agents, particularly for the HPLC–MS approach, as well as luminescent sensors based on nanomaterials [234,235]. The creation of new sorbents for chromatography is of great significance to enable the simultaneous determination of reduced thiols and their disulfides, although research in this area is currently limited.

Emerging trends include the following:

- The continuous advancement of chemiluminometry systems and techniques and enhanced data processing could be the optimal solution for accurately determining the thiol/disulfide ratio in clinical and laboratory diagnostics. In this context, chemiluminometry emerges as a promising method. Notably, individual results obtained with this approach highlight its powerful potential for screening the health status of patients.
- Another notable trend is the increasing number of studies focused on modifying carbon electrodes with various materials, with promise seen in screen-printed electrodes. These ongoing developments hold great potential for enhancing the capabilities and sensitivity of analytical methods for thiol/disulfide ratio assessment.
- Machine learning and chemometric methods will significantly contribute to developing rapid, precise, robust, and easily implementable techniques for determining the thiol/disulfide ratio and classifying big array data for medicine.

## Figures and Tables

**Table 1:** The key constituents of blood plasma involved in redox homeostasis: typical levels in physiologically healthy individuals [25,52].

Substance	*c*, μM			
	Total Concentration *	Reduced Form	Low-Molecular-Weight Disulfide	Protein Disulfide
Human serum albumin (HSA)	527–783	422 ± 52		
Cys	202–281	8.3–10.7	41–63	145–176
Cysteinylglycine (dipeptide)	18.6–35.8	2.0–2.9	4.4–6.8	11–20
Hcys	6.5–11.9	0.17–0.32	1.0–1.2	7.3–10.4
GSH	4.9–7.3	2.0–5.1	0.7–1.6	0.7–1.9
*γ*–Glutamylcysteine	3.1–5.4	0.06		
Hydrogen sulfide	1 × 10^−4^			
Glutathione peroxidase 3	0.5–0.8			
Thioredoxin	(0.07–1) × 10^−3^			
Thioredoxin reductase	3 × 10^−4^			
Glutaredoxin	1.1 × 10^−3^			

* Total concentration=Reduced form+2×Low molecular weight disulfide+Protein disulfide.

**Table 4:** Determination of biothiols by electrochemical methods.

Type of Electrode	Working Electrode Material	Technique	Object	Analyte(s)	LOD, μM	Linear Range, μM	Reference
Unmodified electrodes (hybrid methods)	Au	HPLC coupled with amperometric detection (multiple electrochemical cells 0.4 ÷ 0.85 V vs. Pd)	Rat blood plasma	GSH/GSSG, Cys/Cyss, methionine	0.01–0.09	0.1–1 10–20	[187]
	BDD	HPLC coupled with amperometric detection (*E* = 1.40 V vs. Pd)	Lysed cancer cells	GSH, GSSG	0.001	0.002–1	[188]
	Porous graphite electrodes	HPLC coupled with coulometric detection (dual analytical cell, at 0.35 ÷ 0.88 V)	Blood plasma	Aminothiols and reduced dithiols	(5–50) × 10^−6^	0.1–0.5 50–300	[190]
	Multiple Au microelectrodes	CE with amperometric detection (*E* = 0.42 and 0.48 V vs. Ag/AgCl)	Heparinized blood samples	Cys, Hcys	0.05	0.5–3	[191]
	Au and graphite electrodes	HPLC coupled with electrochemical detection (*E* = 0.600 and 0.750 V vs. Ag/AgCl)	Whole blood	GSH, Cys, *N*-acetylcysteine	–	(0.2–0.5)–8.0	[192]
	NO-selective carbon fiber electrode	Amperometry with ion-selective electrode	Blood serum	*S*-nitrosothiols	5 × 10^−5^	5 × 10^−3^–1	[201]
Electrodes modified with nanomaterials	Carbon-sphere-modified glassy carbon electrode	Amperometry (0.00 V vs. Ag/AgCl)	Human plasma and urine	Total biothiols	0.4	0.2–100	[195]
	Glassy carbon electrode modified with NiO nanoparticles	Amperometry (0.35 V vs. Ag/AgCl)	GSH eye drops	GSH	2	12.5–2300	[196]
	Carbon screen-printed electrode with cytochrome-*c*-anchored Au nanoparticles	Voltammetry	Blood plasma	Hcys	0.3	0.4–700	[202]
	Glassy carbon electrode modified with cobalt phthalocyanine and MWNT	Differential pulse voltammetry	Rat urine and plasma	GSSG, GSH	8 GSSG, 100 GSH	150–7000 (GSSG), 500–3000 (GSH)	[198]
	Glassy carbon electrode modified with poly(caffeic acid) nanocarbon composite	Cyclic voltammetry (ratio of forward and backward peak currents was used)	PBS, pH 7.0	GSH	0.5	0.5–5000	[199]
	Indium tin oxide ITO electrode modified with silver–polydopamine core–shell nanoparticles	Cyclic voltammetry and linear sweep voltammetry	GSH eye drops	Cys	0.02	0.05–300	[208]
	Screen-printed multiwalled carbon nanotube-modified electrode	Square-wave and cyclic voltammetry with catechol as redox mediator	Synthetic saliva, blood plasma, tissue culture media	HCys, GSH	0.9 HCys, 2 GSH	5–20	[209,210]
Immunosensors	AuNPs, graphene, and ionic liquid-modified carbon paste electrode	Differential pulse voltammetry with DNA sensor using thionine-labeled AuNP as an electrochemical probe	Lymphocytic leukemia cell extract	Protein, non-protein thiols	4 × 10^−7^	1 × 10^−6^–0.01	[197]
	AuNPs on the multiwall-based carbon ionic liquid electrode coated with colloidal AuNPs through thiol groups of 1,6-hexanedithiol monolayer as a crosslinker	Impedance spectroscopy with immunosensor (antigen–antibody) using [Fe(CN)_6_]^3−^/^4−^ as redox probe	Human urine and serum	HSA	15.4 ng mL^−1^	0.1–100 µg mL^−1^	[197,200]
Enzymatic biosensors	Glassy carbon electrodes modified with osmium redox polymer and bilirubin oxidase on the cathode and glucose oxidase on the anode	Amperometry in self-powered regime (biofuel cell)	Blood serum	GSH	40	40–2000	[205]
	Glassy carbon electrode modified with graphene oxide and Nafion with covalently immobilized GSH-Px	Differential pulse voltammetry	Hemolyzed erythrocyte	GSH	9 × 10^−4^	0.003–370	[203]
	Pt electrode covered with membranes and immobilized	Amperometric detection of H_2_O_2_ (*E* = 0.65 V vs. Ag/AgCl) or O_2_ (Clark electrode)	Hemolyzed erythrocytes	GSH	-	5–1000	[204]
MOFs and MIPs	Co-metal–organic coordination polymer-modified carbon paste electrode	Constant potential amperometry (*E* = 0.400 V vs. Ag/AgCl)	GSH eye drops	GSH	2.5	25–950	[207]
	Molecularly imprinted polyaniline on indium tin oxide substrate	Differential pulse voltammetry with [Fe(CN)_6_]^3−^/^4−^ as redox probe	PBS, pH 7.0	BSA	0.6 ng mL^−1^	1 × 10^−3^–100 µg mL^−1^	[215]
	Molecularly imprinted polymer film on Au electrode	Electrochemical impedance spectroscopy (EIS) and differential pulse voltammetry (DPV)	Artificial blood serum	HSA	0.8 µg mL^−1^ (EIS) 20 ng mL^−1^ (DPV)	0.8–20 µg mL^−1^ (EIS), 4–80 µg mL^−1^ (DPV)	[218]
	Epitope molecularly imprinted polymer-modified Au-coated quartz crystal	Quartz crystal microbalance (QCM)	Human serum	HSA	0.03 µg mL^−1^	0.05–0.5 µg mL^−1^	[219]

**Table 5:** A review of the methods’ merits.

	Methods	Molecular Spectroscopy	Chromatography	Mass Spectrometry	Electrochemistry
Indicator					
Applications (most important)		Used for measuring thiol concentrations in biological fluids. As for (chemi-)luminescent techniques, it is promising method for high-sensitivity thiol detection.	Employed for the separation and quantification of thiols and disulfides.	Applied for detailed characterization and quantification of thiol compounds. Metabolite analysis.	Employed for real-time monitoring of thiol levels and redox status in blood, etc.
Sample Preparation		Minimal; Requires care in handling to protect thiols from oxidation.	Required; Requires care in handling to protect thiols from oxidation; Often requires derivatization.	Required; Involves complex procedures to stabilize thiols; requires derivatization.	Commonly used for direct or mediated analysis; Requires care in handling to protect thiols from oxidation.
Selectivity and its regulation		Moderate; High in chemiluminescent techniques (CL); Speciation analysis is not supported. CL: detect thiols in complex matrices.	High; Excellent separation capability of thiol species.	Very high; Can adequately distinguish thiol species and their derivatives.	Variable; Can be improved with modified electrodes.
Merits		Simple and cost-effective but may lack selectivity. It is promising for real-time applications.	LOD, though analysis times are longer.	High sensitivity.	Rapid analysis, including in real time.
LOD		As low as 1 μM; As low as 20 nM (for CL).	As low as 1 μM.	In the low-picomolar (pM) range.	ca. 10 nM.

## Data Availability

There are no data associated with this publication.

## Abbreviations

ABD-F	4-(Aminosulfonyl)-7-fluoro-2,1,3-benzoxadiazole
ABEI	*N*-(4-aminobutyl)-*N*-ethylisoluminol
AP-1	Activator protein-1
BDD	Boron-doped diamond
ca.	Circa (about)
CD	Carbon dots
CL	Chemiluminescence
CE	Capillary electrophoresis
*LOD*	Detection limit (also LOD)
*c*_lim_	Determination limit (also LOQ)
CMLT	2-Chloro-1-methylpidinium tetrafluoroborate
CMQT	2-Chloro-1-methylquinoline tetrafluoroborate
Cys	Cysteine
Cys-Gly	Cysteinyl–glycine
DTNB	5,5′-Dithiobis-(2-nitrobenzoic acid), Ellman’s reagent
E_h_	Redox potential value of the biological environment, e.g., plasma-reducing potential
FT–Raman	Fourier-transform Raman spectroscopy
GC-MS	Gas chromatography–mass spectrometry
Glu-Cys	Glutamyl–cysteine
GR	Glutathione reductase
GSH	Glutathione
GS-TNB	Glutathione adduct of TNB
Hcys	Homocysteine
HPLC–FL	High-performance liquid chromatography with fluorimetric detection
HPLC-MS	High-performance liquid chromatography–mass spectrometry
HPLC-UV	High-performance liquid chromatography with ultraviolet detection
HSA	Human serum albumin
IAM	Iodoacetamide
IPCF	Isopropyl chloroformate
FIA-CL	Chemiluminometric Flow Injection Analysis
MAPK	Mitogen-activated protein kinase
mBrB	Monobromobiman
MIPs	Molecularly imprinted polymers
MOF	Metal–organic frameworks
MIPBO	5-Methyl-(2-(*m*-iodoacetylaminophenyl)benzoxazole
MMTS	Methylmethantiosulfonate
MSTFA-TMCS	2,2,2-Trifluoro-N-methyl-N-(trimethylsilyl)-acetamide + 1% Chlorotrimethylsilane
MSTP	4-(5-Methanesulfonyl-[1,2,3,4]tetrazol-1-yl)phenol
MWNT	Multiwalled carbon nanotubes
NEM	*N*-ethylmaleimide
NF-kB	Nuclear Factor Kappa B
nLC–HR-MS/MS	Nanoflow liquid chromatography and high-resolution tandem mass spectrometry
NO	Nitric oxide
NPM	*N*-phenylmaleimide
ONOO^−^	Peroxynitrite
POOH	Hydroperoxide
PTP	Protein tyrosine phosphatase
qBBr	Monobromo(trimethylammonio)bimane
RNS	Reactive nitrogen species
ROS	Reactive oxygen species
RS^•^	Reactive thiol radical
R-SH	Reduced form(s) of thiol(s)
R-SO_2_H	Sulfinic acid
R-SO_3_H	Sulfonic acid
RSOH	Sulfenic acid
RSOO^•^	Peroxyl radical
R-SS	Intramolecular disulfide
R-S-S-R	Intermolecular disulfide
R-S-S-R′	Oxidized (disulfide) form(s) of thiol(s)
SBD-F	7-Fluorobenzo-2-oxa-1,3-diazole-4-sulfonate
SDS-PAGE	Sodium dodecyl sulfate–polyacrylamide gel electrophoresis
SERS	Surface-enhanced Raman scattering
TCEP	Tris(2-carboxyethyl)phosphine
THz–TDS	Terahertz time-domain spectroscopy
TNB	5-Thio-2-nitrobenzoic acid
UHPLC	Ultra-high-performance liquid chromatography
^•^O_2_^−^	Superoxide anion radical
2-VP	2-Vinylpyridine
